# Platelet-Function-Monitoring-Guided Therapy After Emergent Carotid Artery Stenting

**DOI:** 10.3390/jcm13226690

**Published:** 2024-11-07

**Authors:** Magnus Peter Brammer Kreiberg, Nicolaj Grønbæk Laugesen, Andreas Hjelm Brandt, Trine Stavngaard, Joan Højgaard, Thomas Truelsen

**Affiliations:** 1 Cerebrovascular Research Unit Rigshospitalet, Department of Neurology, Copenhagen University Hospital, Blegdamsvej 9, 2100 Copenhagen, Denmark; 2 Department of Radiology, Rigshospitalet, Copenhagen University Hospital, Blegdamsvej 9, 2100 Copenhagen, Denmark; 3 Faculty of Health Sciences, University of Copenhagen, Blegdamsvej 3B, 2200 Copenhagen, Denmark

**Keywords:** stroke, thrombectomy, carotid artery stenting, clopidogrel resistance, platelet function monitoring

## Abstract

**Background/Objectives:** Antiplatelet therapy after emergent carotid stenting (eCAS) represents a challenge in balancing the risk of intracerebral hemorrhages (ICHs) and in-stent thrombosis (IST). Post-procedural platelet function monitoring may guide antiplatelet therapy and could potentially improve outcomes due to fewer post-procedural complications. **Methods:** Consecutive eCAS patients (2019–2021) were included in a single-center retrospective observational study. Patients treated with eCAS received peri-procedural eptifibatide followed by dual antiplatelet treatment with aspirin and clopidogrel. The effect of platelet ADP inhibition by clopidogrel was monitored using the Multiplate^®^ Analyzer (Roche). Clopidogrel non-responders were changed to ticagrelor treatment. The primary outcome was defined as a favorable outcome at 90 days using the modified Rankin Scale (mRS) of 0–2 versus 3–6. Safety outcomes included ICH, IST, and mortality. Data were analyzed and compared in clopidogrel- and ticagrelor-treated patients using Fischer’s exact test and multivariate logistic regression. **Results:** A total of 105 patients had eCAS, and 28 patients (27%) were clopidogrel non-responders and were changed to treatment with ticagrelor. The favorable outcome was more frequent in ticagrelor-treated patients, 23 (82%), than in clopidogrel-treated patients, 44 (57%), *p* = 0.036. Numerically, ICH, IST, and mortality were more frequent in clopidogrel-treated patients, but none of the differences were statistically significant. In multivariate analyses, ticagrelor treatment was significantly associated with the favorable outcome, OR = 3.89 (95% CI: 1.09–13.86), *p* = 0.036. **Conclusions:** One in four eCAS patients were clopidogrel non-responders. This study suggests that personalized antiplatelet treatment therapy was safe, and that changing treatment to ticagrelor in clopidogrel non-responders was associated with better outcomes in eCAS patients.

## 1. Introduction

Acute ischemic stroke (AIS) is a major cause of death and adult-acquired disability worldwide [1]. While only every fourth patient with AIS has a large vessel occlusion, these patients contribute disproportionately to stroke fatalities and post-stroke disability [2]. However, clinical trials have shown mechanical thrombectomy (MT) to be a safe and effective treatment of AIS with large vessel occlusions [3]. Following these trials, the MT indication has broadened extensively and is now recommended in patients beyond six hours from onset [4], patients with large infarcts [5], and patients with basilar artery occlusions [6]. However, no randomized controlled trials have studied specifically the management of tandem occlusions with simultaneous lesions of the extracranial internal carotid artery (ICA) and an intracranial vessel occlusion [7,8,9]. This represents a challenge as 15–30% of patients treated with MT have tandem lesions [3,9,10], and the deployment of emergent carotid artery stenting (eCAS) in tandem lesions has demonstrated high rates of successful reperfusion and good clinical outcome [10,11,12]. However, whereas treatment with eCAS is part of standard care [13,14], it is associated with a risk of both in-stent thrombosis (IST), ranging from 5 to 18% [15,16,17,18,19], and symptomatic intracerebral hemorrhage (sICH), ranging between 4.6 and 9% [12,20,21]. The risk of IST is managed by the acute administration of platelet inhibitors, which may increase the risk of sICH, but there are currently no recommendations on the administration of platelet inhibitors after eCAS is present.

Platelet inhibitors acetylsalicylic-acid (ASA) and clopidogrel exert their effect through interaction with the platelet arachidonic acid receptor (ASPI) and the adenosine diphosphate receptor (ADP), respectively. Previous studies have shown that up to every fourth patient has a high platelet reactivity despite treatment with clopidogrel [22], and these patients may have a higher risk of IST [23]. Changing the treatment to another ADP-receptor antagonist such as ticagrelor may, on the other hand, increase the risk of ICH [23,24]. The post-procedural monitoring of patients’ individual platelet inhibition could enable the optimization of balancing the risk of IST and ICH [23,24,25,26,27].

The aim of this study was to evaluate functional outcome and the occurrence of IST and ICH in eCAS patients where post-procedural antiplatelet treatment was guided by using a Multiplate^®^ Analyzer (Roche).

## 2. Methods

Consecutive patients undergoing MT at a single comprehensive stroke center were enrolled from January 2019 to December 2021, in a retrospective observational study. The stroke center had a catchment area of 2.6 million inhabitants (2020 census) and has been described elsewhere [28]. Adult patients undergoing MT were included in this study if treated with eCAS within the study period and were excluded if arterial stenting was performed as a planned subacute procedure. Patient eligibility for MT was an interdisciplinary decision made by the vascular neurologist and the neurointerventionalist on call based on imaging assessment and clinical symptoms [13]. The procedure was performed at the discretion of the performing neurointerventionalist, including the use of eCAS, which was performed in patients with either symptomatic stenosis, dissection, or occlusion of the ICA. For eCAS patients, peri-procedural treatment to prevent IST was mandatory, regardless of pre-treatment with intravenous thrombolysis (IVT). Local guidelines were followed throughout the observation period for periprocedural treatment, with the administration of eptifibatide, which is a glycoprotein IIb/IIIa inhibitor, at 180 µg/kg body weight and a loading dose of ASA 300–500 mg followed by ASA 75–150 mg BID and a clopidogrel loading dose of 150–300 mg followed by 75–150 mg QD.

Following the MT procedure, platelet function was monitored for each patient individually using the Multiplate^®^ Analyzer (Roche Diagnostics, Bern, Switzerland). Platelet function is expressed as TRAP-test (thrombin-receptor activating peptide (TRAP)-6), ASPI-test, and ADP-test where the analysis measured the impedance increase in relation to agonist-induced platelet aggregation on the specific receptors [29]. Sufficient platelet inhibition was defined as a minimum 50% ADP (clopidogrel effect) and ASPI (aspirin effect) inhibition relative to the normal TRAP value range of 92–151 [29,30]. Patients with insufficient ADP inhibition < 50% after the initial post-procedural clopidogrel loading dose or first administration of the maintenance treatment were defined as clopidogrel non-responders and changed from clopidogrel to ticagrelor treatment with a loading dose of ticagrelor 180 mg followed by 90 mg BID.

### 2.1. Outcome

The primary outcome was a functional outcome at 90 days using the modified Rankin Scale (mRS). A favorable outcome was defined as an mRS of 0 to 2, which describes patients with no symptoms ranging to some residual symptoms but independent in activities of daily living, while a non-favorable outcome was an mRS of 3 to 6, which ranges from the need of assistance for daily living to death. Safety outcomes included IST, sICH, all ICH, and all-cause mortality.

In-stent thrombosis was defined as any post-procedural IST that occurred at any time during the 3 month follow-up, which included a routine CTA/MRA at 3 months. A post-procedural dual-energy CT-scan was carried out after 24 h in all patients. The presence of ICH was retrospectively classified according to the Heidelberg and European Cooperate Acute Stroke Study (ECASS) II classifications by a neuroradiologist [31]. The classification differed between hemorrhagic transformation with hematoma occupying <30% of the infarcted tissue type 1, occupying >30% of infarcted tissue type 2, and intracerebral hemorrhage outside the infarcted tissue or extracranial hemorrhage including subarachnoid hemorrhage type 3. Any ICH was a secondary outcome, defined as any type of hemorrhage according to the Heidelberg classification, including hemorrhagic transformation, which was identified at the 24 h dual-energy CT-scan. Symptomatic intracerebral hemorrhage (sICH) was defined as an increase of >3 points on the National Institute of Health Stroke Scale (NIHSS), and CT or MRI verified ICH in the relevant cerebral area.

### 2.2. Data Variables

Clinical and demographical data of patients were recorded, including pre-stroke mRS, medical history with cardiovascular risk factors, use of antiplatelet or anticoagulative treatment, acute treatment with intravenous thrombolysis (IVT), and stroke severity using the National Institute of Health Stroke Scale (NIHSS). Early ischemic changes on the pre-procedural imaging were reviewed retrospectively by a neuroradiologist using the Alberta Stroke Program Early Computed Tomography (ASPECT) score [32]. Post-procedural reperfusion was assessed using the modified Thrombolysis in Cerebral Infarction (mTICI) classification by the performing neurointerventionalist [33]. An mTICI score of 0 was defined as no reperfusion and a score of 3 as full reperfusion. An mTICI score of 2b-3 was regarded as successful reperfusion. Clinical follow-up after 3 months included the registration of functional status using the modified Rankin Scale (mRS), where a score 0–2 was defined as a favorable outcome.

### 2.3. Statistics

Continuous variables are presented as medians with the interquartile range (IQR) and categorical variables are presented with frequencies and percentages. For comparing baseline characteristics and outcomes, Fischer’s exact test was used for categorical variables and Mann–Whitney U for numerical variables. Multivariate logistic regression was used to test the association of ticagrelor with favorable outcomes at 90 days when adjusted for age (cont.), sex (male), baseline NIHSS (cont.), ASPECT-score (cont.), and pre-stroke mRS (0 vs. >0). The level of statistical significance was defined as *p* < 0.05.

## 3. Results

In the study period, 920 patients with stroke underwent emergent endovascular therapy, of which 105 (11.4%) were treated with eCAS. Twenty-eight patients (27%) were clopidogrel non-responders and were changed to ticagrelor (Table 1).

The median age of ticagrelor-treated patients was 64 years (IQR 53–76) compared to 67 (IQR 57–76) of clopidogrel-treated patients. Patients were predominantly male, 68% and 66%, in ticagrelor- and clopidogrel-treated patients, respectively, and most patients were treated with IVT, 60% and 61%. The median ASPECT-score was 9 in both groups. The median NIHSS at baseline was 16 (IQR 10–21) and 15 (IQR 9–20), and at 24 h, 6 (IQR 1–12) and 8 (IQR 4–15), in ticagrelor- and clopidogrel-treated patients, respectively. Time from onset to groin puncture was higher in the clopidogrel group, with a median of 220 min (IQR 131–163), compared to the ticagrelor group, with a median of 200 min (IQR 129–442), as was the procedural time, with a median of 55 min (IQR 38–103) compared to 45 (IQR 35–73). In the clopidogrel group, tandem occlusions were the most frequent pathology (47%), while ticagrelor patients more often had isolated ICA occlusions (57%).

A favorable outcome was more common in the ticagrelor-treated patients (n = 23, 82%) than in the clopidogrel-treated patients (n = 44, 57%) (Table 2). Safety variables were not statistically different between the two groups; however, numerically more complications occurred in the clopidogrel group than the ticagrelor group: sICH (n = 5, 6.5% vs. n = 1, 3.6%), any ICH (n = 18, 23% vs. n = 4, 14%), in-stent thrombosis (n = 2, 2.6% vs. n = 0), and death (n = 6, 7.8% vs. n = 1, 3.6%), respectively.

Substitution with ticagrelor in clopidogrel non-responders was significantly associated with a favorable outcome in univariate logistic regression, OR 3.24 (95% CI: 1.11–9.46, *p* = 0.04). After adjustment for age, sex, pre-stroke mRS, NIHSS, and ASPECTS, substitution with ticagrelor remained significantly associated with a favorable outcome, OR 3.90 (95% CI: 1.09–13.86, *p* = 0.04) (Table 3).

## 4. Discussion

Six-in-ten stroke patients had a favorable functional outcome following eCAS with antiplatelet treatment guided by platelet function monitoring. One in four patients undergoing eCAS were clopidogrel non-responders and antiplatelet treatment was changed to ticagrelor. A favorable outcome was observed statistically significant more often in patients receiving ticagrelor, while differences in sICH, any ICH, and IST were not significant, though numerically more common in the clopidogrel group.

Treatment with platelet-inhibitors have been the subject of several previous studies, reflecting the shortage of data for preventing post-procedural complications and a lack of randomized controlled trials for a firm treatment strategy. In a recent study of 300 patients undergoing eCAS and treated with acetylsalicylic acid 100 mg + clopidogrel 75 mg, o.d., those who had high-on-treatment platelet reactivity measured using different assays were also at higher risk of major adverse cardiovascular events during the 5.8 years median follow-up [34]. Only two patients experienced stent-thrombosis, suggesting that increased platelet reactivity may be a cause of increased morbidity and mortality beyond the 3 months of observations we had in this study. Other platelet regiments have been tested in eCAS patients such as tirofiban, a glycoprotein IIb/IIIa inhibitor, used as a single platelet inhibitor [35] showing better functional outcome and a lower risk of intracranial hemorrhage at 90 days when compared to combined treatment with acetylsalicylic acid. Our study is an extension of such previous studies as we measured platelet inhibition activity and changed treatment based on pre-defined criteria, showing a favorable outcome predominantly driven by a decrease in any intra-cranial hemorrhage.

Previous studies of sICH rates show that eCAS varies from 4 to 8% [36,37,38,39], which is comparable to the 6% in the total population of our data. For this study, the sICH definition from ECASS II has a broader definition of sICH compared to other classifications. However, “any ICH” was also less common in ticagrelor-treated patients, suggesting that the change in platelet inhibition was safe, although the difference did not reach statistical significance, probably due to the limited sample size.

Only two cases of post-procedural IST (2%) were observed, and though both events occurred in the clopidogrel group, the low frequency constrained our ability to make any conclusions on factors contributing to its occurrence. However, two recent observational cohorts reported IST in 11–22% of patients and did not use platelet function monitoring to guide antiplatelet treatment [36,37]. Both studies administered dual antiplatelet treatment during the procedure and continued treatment after imaging at 24 h, while one of the cohorts used procedural eptifibatide in 10% of patients in whom no IST was observed [36]. It should be noted that the use of eptifibatide has been suspected of resulting in a higher risk of sICH compared to procedural dual antiplatelets, though not significantly (11% vs. 7%, respectively) [40].

Ticagrelor in monotherapy or combined with ASA was not superior to ASA after minor stroke in clinical trials [41,42]. Indeed, the combination of ASA and ticagrelor increases the risk of ICH compared to ASA alone [41]. However, in clopidogrel non-responders, ticagrelor reduced the risk of recurrent stroke while not increasing the risk of ICH compared to clopidogrel [43]. These trials suggest that the most optimal platelet-inhibition treatment may be a personalized strategy depending on individual risk factors and responder status. In the presented study, clopidogrel non-responders were substituted with ticagrelor based on platelet reactivity, which was associated with improved functional outcome. Due to the study design, the potential superiority of this treatment regimen cannot be addressed. A randomized clinical trial on antiplatelet treatment following eCAS is still warranted. Currently, four trials are recruiting stroke patients with tandem lesions randomized to either MT with eCAS or without eCAS (ClinicalTrials.gov ID: NCT03978988, NCT05611242, NCT05902000, and NCT04261478). However, according to the study descriptions, none of the trials will take platelet reactivity status into account. This could potentially diminish a benefit from eCAS as both IST and sICH would worsen functional outcomes and the risk would be mitigated if considering platelet reactivity.

## 5. Strengths and Limitations

Our study has several strengths including an adherence to pre-defined criteria for treatment with antiplatelets and a test for platelet function. Furthermore, due to universal health care coverage, the risk of socio-economic bias is limited, representing the standard eCAS patient population in our catchment area. In addition, this study did not receive external funding and the results and conclusions are therefore only representative of those of the authors. Furthermore, we used a consecutive study design with the inclusion of all patients treated with eCAS during the observation period.

However, our study also has several limitations of which one relates to the time for ticagrelor substitution, which could be delayed as much as 24 h post procedure. Delayed substitution typically occurred in patients with a prolonged effect of eptifibatide administered during the procedure, which was evaluated with platelet function monitoring also using Multiplate^®^ Analyzer [29]. The delayed substitution introduced a risk of immortal time bias, as early fatalities or clinical deterioration leading to withdrawal of care would potentially not be evaluated for antiplatelet treatment substitution. As a result, the potential bias may overestimate any benefit of ticagrelor over clopidogrel. Also, our study is a single-center retrospective observational study without a control group, and therefore we cannot draw any conclusions on the superiority of one regime over another. A further limitation of this study is the variability by using different assays and equipment available for testing platelet reactivity; although multiple devices exist, e.g., VerifyNow and VASP [34], our study was limited to the use of Multiplate^®^ Analyzer [29].

## 6. Conclusions

In this observational cohort, six in ten patients achieved a favorable functional outcome after ischemic stroke with eCAS with platelet function monitoring to guide antiplatelet treatment, while complications were rare. One in four patients were clopidogrel non-responders and were substituted with ticagrelor with numerically fewer post-procedural complications and significantly better outcomes at 3 months. These results suggest that antiplatelet treatment guided by function monitoring should be considered safe and that ticagrelor is a safe alternative in clopidogrel non-responders.

## Figures and Tables

**Table 1:** Baseline characteristics of eCAS patients according to antiplatelet treatment.

	Clopidogrel (n = 77, 73%)	Ticagrelor (n = 28, 27%)	*p*-Value
Age, years, median (IQR)	67 (57–76)	64 (53–76)	0.712
Sex, male	51 (66%)	19 (68%)	0.536
Pre-stroke mRS of 0	63 (82%)	25 (89%)	0.276
NIHSS at baseline, median (IQR)	16 (10–21)	15 (9–20)	0.758
NIHSS at 24 h, median (IQR)	8 (4–15)	6 (1–12)	0.053
Intravenous thrombolysis	46 (60%)	17 (61%)	0.556
ASPECTS, median (IQR)	9 (7–10)	9 (8–10)	0.668
Medical history:			
Diabetes mellitus	11 (14%)	5 (18%)	0.430
Atrial fibrillation	6 (8%)	1 (4%)	0.397
Ischemic heart disease	5 (7%)	3 (11%)	0.362
Hypertension	38 (49%)	18 (64%)	0.128
Prior stroke or TIA	12 (16%)	3 (11%)	0.415
Peripheral arterial disease	5 (7%)	2 (7%)	0.603
Medical treatment at admission:			
Platelet inhibitors	23 (30%)	8 (29%)	0.551
Anticoagulation treatment	1 (1.3%)	0 (0%)	0.733
Statins	25 (33%)	7 (25%)	0.315
Procedural measures:			
Time from onset to groin puncture, minutes	220 (131–363)	200 (129–442)	0.736
Time from groin puncture to end of procedure, minutes	55 (38–103)	45 (35–73)	0.099
General anesthesia	73 (95%)	27 (96%)	0.597
Successful reperfusion (mTICI 2b-3)	69 (90%)	27 (96%)	0.473
Occlusion site:			0.237
Tandem	36 (47%)	8 (29%)	
Isolated ICA	34 (44%)	16 (57%)	
T occlusion	7 (9%)	4 (14%)	

Values are n (frequency) unless otherwise stated. Abbreviations: eCAS = emergent carotid artery stenting, MT = mechanical thrombectomy, ICA = internal carotid artery, mRS = modified Rankin Scale, NIHSS = National Institute of Health Stroke Scale, ASPECTS = Alberta stroke program early CT score, TIA = transient ischemic attack, mTICI = modified treatment in cerebral ischemia. T occlusion is an occlusion of the terminal segment of the ICA and the first segments of the ipsilateral anterior and middle cerebral arteries.

**Table 2:** Primary and safety outcomes.

	Clopidogrel (n = 77, 73%)	Ticagrelor (n = 28, 27%)	*p*-Value
Favorable outcome at 90 days (mRS 0–2) *	44 (57%)	23 (82%)	0.036
In-stent thrombosis	2 (2.6%)	0 (0%)	1.000
Any ICH	18 (23%)	4 (14%)	0.420
sICH	5 (6.5%)	1 (3.6%)	1.000
Death within 90 days *	6 (7.8%)	1 (3.6%)	0.385

Values are n (%). * Two patients of the eCAS group were lost to follow-up at 90 days. Abbreviations: ICH = symptomatic/asymptomatic intracerebral hemorrhage, sICH = symptomatic intracerebral hemorrhage, mRS = modified Rankin Scale, NA = not applicable. P = Fischer’s exact test.

**Table 3:** Univariate and multivariate logistic regression of favorable outcome.

	Univariate	*p*	Multivariate	*p*
Age, cont.	0.929 (0.892–0.969)	<0.001	0.931 (0.886–0.978)	0.004
Sex, male	0.736 (0.303–1.786)	0.498	1.149 (0.393–3.361)	0.800
Ticagrelor treatment	3.241 (1.111–9.456)	0.031	3.888 (1.091–13.860)	0.036
Pre-stroke mRS 0	7.875 (2.312–26.818)	<0.001	0.455 (0.196–1.056)	0.067
NIHSS baseline, cont.	0.951 (0.900–1.006)	0.078	0.942 (0.882–1.007)	0.078
ASPECTS, cont.	1.214 (0.943–1.564)	0.132	1.245 (0.923–1.680)	0.151

Dependent variable was favorable outcome defined as mRS 0–2 at 90 days. Abbreviations: mRS—modified Rankin Scale, NIHSS—National Institute of Health Stroke Scale, ASPECTS—Alberta Stroke Programme Early CT.

## Data Availability

Approvals given for the conduction of this study did not grant permission to make individual patient data available. Statistical analyses can be made available upon reasonable request to the corresponding author.
